# Developing a strategy for computational lab skills training through Software and Data Carpentry: Experiences from the ELIXIR Pilot action

**DOI:** 10.12688/f1000research.11718.1

**Published:** 2017-07-03

**Authors:** Aleksandra Pawlik, Celia W.G. van Gelder, Aleksandra Nenadic, Patricia M. Palagi, Eija Korpelainen, Philip Lijnzaad, Diana Marek, Susanna-Assunta Sansone, John Hancock, Carole Goble

**Affiliations:** 1 New Zealand eScience Infrastructure, Auckland, 1061, New Zealand; 2 ELIXIR-Netherlands, Dutch Techcentre for Life Sciences (DTL), Utrecht, 3511, Netherlands; 3 ELIXIR-UK, Software Sustainability Institute UK, School of Computer Science, University of Manchester, Manchester, M13 9PL, UK; 4 ELIXIR-Switzerland, SIB Swiss Institute of Bioinformatics, Lausanne, 1015, Switzerland; 5 ELIXIR-Finland, CSC - IT Center for Science, Espoo, 02101, Finland; 6 Princess Máxima Center for Pediatric Oncology, Utrecht, 3584, Netherlands; 7 ELIXIR-UK, Oxford e-Research Centre, Oxford University, Oxford, OX1 3QG, UK; 8 ELIXIR-UK, Earlham Institute, Norwich, NR4 7UZ, UK

**Keywords:** Life sciences, bioinformatics, training, IT and computational skills, data analysis, capacity building, software carpentry, data carpentry

## Abstract

Quality training in computational skills for life scientists is essential to allow them to deliver robust, reproducible and cutting-edge research. A pan-European bioinformatics programme, ELIXIR, has adopted a well-established and progressive programme of computational lab and data skills training from Software and Data Carpentry, aimed at increasing the number of skilled life scientists and building a sustainable training community in this field. This article describes the Pilot action, which introduced the Carpentry training model to the ELIXIR community.

## Introduction

As research in life sciences develops on the fast track, the need for federated resources and infrastructure, and coordinating activities supporting researchers, is becoming increasingly important. ELIXIR is a European research infrastructure with a mission to manage and safeguard the increasing volume of data generated by life science research. It coordinates and sustains bioinformatics resources across its member states and help researchers to more easily find, analyse, share data and exchange biological data. ELIXIR follows a Hub and Nodes model, with a single Hub based in Hinxton, United Kingdom, and a growing number of Nodes located at centres of excellence throughout Europe. At the time of writing, ELIXIR has 20 national Nodes, and European Bioinformatics Institute (EMBL-EBI; co-located with the Hub), working as a separate Node.

Providing necessary training to researchers to tackle emerging research data manipulation and computing issues is of key priority and one of ELIXIR’s main missions ( van Gelder *et al*., 2016).

### ELIXIR Software and Data Carpentry Pilot action

The ELIXIR Hub strongly encourages interactions and collaborations between the Nodes. Such interactions are supported by short-term Pilot actions ^1^ that are funded by ELIXIR. The goal of these projects is to “tackle major European challenges in life science data access, high-performance computing and the interoperability of public biological and biomedical data resources”. Pilot actions usually involve several ELIXIR Nodes to build, test and demonstrate the value of the distributed infrastructure.

One of these collaborative activities is providing training in essential skills for life science researchers throughout ELIXIR. In part, these skills encompass computational and digital data manipulation and analysis skills. Even though there are a lot of materials available to teach these topics, they are scattered, hard to discover and access, or assume too much prior knowledge. Contrarily, there is a lack of training material in biocuration and for the development of content standards (terminologies, minimum information checklists, exchange formats) and their use (e.g. in annotation tools). Nevertheless, funders and researchers increasingly call for enhanced, standards-driven experimental annotation at the source, and for data sharing to maximise data reproducibility and reuse, in order to drive science and scientific discoveries ( Wilkinson *et al*., 2016).

Training activities are one of the main focus areas of the ELIXIR UK Node, and the Training Theme that it leads. In 2014, the UK Node, in collaboration with and support from ELIXIR Finland, ELIXIR Netherlands, ELIXIR Switzerland and ELIXIR Sweden, proposed a Pilot action for “Working up and building the foundation for Data Carpentry and Software Carpentry within ELIXIR” to tackle this evident training gap.

The Pilot project had several overarching objectives. The first was to launch the Carpentry initiatives in the ELIXIR community and leverage the work of the Software Carpentry (SWC) and Data Carpentry (DC) initiatives from the US and Canada by reusing their existing training materials and well-tested, successful and popular model of hands-on teaching. Secondly, we wanted to tap into the international community built for years around the Carpentries, which included experienced researchers and trainers in life sciences who have been delivering and developing training on a regular basis. In the long run, the goal was to develop a self-sustainable pool of professional Carpentry instructors within ELIXIR who would deliver training across the Nodes, by incorporating the Instructor Training course, developed by the Carpentries. Finally, we wanted to join and help grow this international community by contributing the newly trained instructors, as well as further developing and adapting materials in the life sciences domain.

### Software and Data Carpentry

SWC is an international collaboration aimed at teaching researchers (without prior knowledge or training in IT) basic software development skills. This initiative is very well established internationally and has been running since 1998.

SWC training courses are highly-interactive two-day workshops that give researchers training in essential software development skills, but in the context of how they contribute to improving research productivity and help produce robust and reproducible science. In other words, they look beyond **just teaching people to code** (i.e. syntax of a language or commands they can run) - teaching people **how to code** and **coding best practices** is as important ( Wilson, 2016).

The materials used for both Carpentry workshops are openly available and developed by the community of experts and experienced instructors. The way workshops are delivered has been devised and perfected over the years of practice and is based on pedagogical approaches, taking into account learners’ backgrounds, reducing cognitive load, and giving and receiving constructive feedback. They employ techniques such as live coding, working in pairs and peer learning to make the training the most effective.

SWC training courses cover four core topics:

• task automation using command line to help with repeating common tasks,
• structured code development, so that scientists produce code that is readable, testable, sustainable and reusable (demonstrated using either Python or R),
• version control for backing up, collaborating and sharing code, and
• introduction to structuring data using SQL and preparing it for further processing.

DC started in 2015 as a separate programme inspired by SWC. Both programmes maintain close ties, which help to build and share a community of practice among the instructors and expand the base of teaching materials and available trainers. DC aims to teach the skills that will enable researchers to be more effective and productive in working with data. As in SWC, teaching is delivered through intensive two-day workshops.

Contrary to SWC, DC designs the workshops to fit into needs of particular domains (e.g. life sciences, social sciences, digital humanities and library carpentries, etc.). The core DC workshop curriculum covers topics such as:

• caveats and best practices of working with spreadsheets for data organisation
• data reading/processing/manipulation/visualisation with R or Python,
• introduction to structuring data using SQL and preparing it for further processing, and
• introduction to OpenRefine for data cleaning.

The instructors delivering the material are members of the SWC/DC instructor network and have completed training designed to prepare them to use “the Carpentry way” of teaching the skills for effective and productive work with research data. All instructors are volunteers and do not get remuneration for the workshops they teach - they do it because of their love of teaching or because they want to give back to the community. For that reason growing a large pool of instructors (primarily peer-researchers) allows for responding to the massive demand for the workshops.

Both Carpentries are aimed at providing researchers with the essential lab skills for computational science. They do this by focusing on the core skills and making sure that the best practices are passed on in a useful and effective way. Carpentries do not aim to teach audiences specific technical aspects of research - such an audience requires different training (within ELIXIR this type of training is covered by the Train the Developers Programme ( van Gelder *et al.*, 2016)). However, SWC/DC can act as an important connector between researchers and service providers, allowing both sides to communicate better and work more effectively.

## Pilot action overview and goals

The Pilot action was delivered between end of the March 2015 and January 2016, and its goals were as follows:

1. introduce the SWC/DC workshops model in ELIXIR Nodes and expand the number of organisations in the ELIXIR community capable of organizing SWC/DC events, as well as expand the SWC/DC global training network;
2. introduce the SWC/DC material development model in ELIXIR Nodes and improve existing materials with ELIXIR-relevant training (during the dedicated material creation hackathons);
3. build a pool of certified SWC/DC instructors within ELIXIR Nodes to create a self-sustainable training community.

We aimed to train as many researchers within the budget and to familiarise ELIXIR training coordinators and the training community from the Nodes with the Carpentry model of teaching. In addition, to introduce them to the ways of how to expand existing and develop new SWC/DC life sciences training materials so they can continue to carry on this practice in their Nodes.

ELIXIR Nodes would become empowered to run Carpentry events, and be able to contribute to both initiatives by becoming a part of the vibrant international SWC/DC community and expand the SWC/DC training network. One of their key contributions would be the collaborative development and improvement of training materials. The contents need to be updated and expanded depending on the community needs, and ELIXIR member organisations are important representatives of the life sciences community.

The first step in material development component was identifying the SWC/DC materials that needed further work and development. The new materials for life sciences would then be assessed through test runs in consecutive Carpentry workshops piloted by the Nodes. The assessment of the outcomes was planned through follow-up surveys and interviews to determine what people actually would adopt and the impact it would have on their research. Finally, this would lead to adopting the new content for regular teaching.

The long-term goal was focused on capacity building in ELIXIR - not just training researchers, but growing a pool of certified instructors and a self-sustainable training community. In order to ensure the quality of teaching at the SWC/DC workshops, at least one of the instructors needs to be an officially certified Carpentry instructor. Certification is obtained through completing the Carpentry Instructor Training course. By training instructors at different ELIXIR Nodes, the Pilot action helped these Nodes to evolve towards being able to run the workshops independently. The teaching methods and techniques discussed at the instructor training are also applicable for training in other topics. Therefore, this event contributed to the overall training capacity building of the Nodes.

## Pilot action delivery

With some SWC/DC workshops already happening, mainly within the ELIXIR UK Node, we had an opportunity to demonstrate the relevance of this type of training for life sciences. In the UK, the Carpentry workshops were coordinated by the Software Sustainability Institute (SSI). The close collaboration between the UK Node and the SSI substantially facilitated the delivery of the Pilot - one of the key people involved in writing the proposal and delivering the Pilot was the training lead at the SSI at the time, and the deputy head of the UK Node was a co-investigator at the SSI. The Pilot action was planned to last for 18 months, but we managed to deliver all tasks within the first 12 months.

### Outreach

The delivery of the Pilot action was coordinated by the ELIXIR UK Node and started in late 2014 with outreach and engagement to ensure broad participation of the Nodes in the planned events. In order to streamline communication with other Nodes, we sent a request for volunteers (one per Node) to step in and become a SWC/DC Coordinator for their Nodes. The information about the planned Pilot activities would be passed on to the Coordinators who would then disseminate it within their Node. This was particularly important at the beginning of the Pilot, as SWC/DC workshops were relatively unknown among ELIXIR research organisations in 2014.

Reaching out to find the Coordinators was an iterative process. A number of people in ELIXIR were actively involved. We posted the call for Coordinators on the ELIXIR UK, as well as the main ELIXIR websites. The ELIXIR Training Coordinator Group (TrCG) was engaged from the very beginning and helped circulate announcements and facilitate communication with the Node members.

As a result, volunteers from 10 Nodes (out of 17 at the time) stepped in. Most of them did not know much about the SWC/DC workshops, but they all had strong interest in teaching. The call for Carpentry Coordinators specified that ideally their work responsibilities should be related to training so that the effort related to coordination would align with their regular responsibilities.

### Workshops and hackathons

The Pilot was delivered as follows. Firstly, we ran four-day events that combined training material creation hackathon (two days) with a regular train-the-researcher workshop (two days). We ran two of these combined events - one in Finland and one in the Netherlands. These were then followed by an Instructor Training event, aimed at selected participants from the first two events who showed the most interest and enthusiasm about the programme and were willing to become instructors themselves.

The material creation hackathons consisted of two parts: the first one was to introduce the idea and running of SWC/DC workshops, curriculum and model of training; the second part was focused on improving existing and developing new training materials specifically focused on life sciences and its various sub-domains. Through these hackathons, we actually wanted to demonstrate another distinctive feature of SWC/DC - collaborative material development. All SWC and DC training materials, websites and other documents, are developed and shared by the community via GitHub. This approach has proven to be very successful, allowing for maximum inclusivity and production of high quality training materials avoiding redundancy in contents ( Wilson *et al*., 2014; Wilson *et al*., 2016). As part of the hackathons, we wanted to train people to become proficient in this method of working, as well as create more materials.

***Combined hackathon and workshop in Finland*.** The first combined event was held in Helsinki, Finland, in March 2015, hosted by ELIXIR Finland at the CSC IT Centre for Science.

The training material creation hackathon was advertised among the Nodes with the idea of bringing together 15 participants representing as many Nodes as possible to ensure wide representation and dissemination. Eventually 12 participants from 11 Nodes were present at the material creation hackathon (the first two days of the event).

The material development covered during the hackathon included:

• development of the dplyr module for the R lessons;
• development of next generation sequencing data analysis lessons;
• development of RNAseq data analysis using BRB Digital Gene Expression;
• improvements on the shell lessons;
• improvement of the spreadsheet lessons.

The facilitators at the hackathon were two experienced Carpentry instructors - one of which was also among the Pilot proposers and a member of the DC Steering Committee at the time. After leading the hackathon, they also taught at the DC workshop that immediately followed (the last two days of the event). The workshop itself was attended by 29 researchers from the life sciences domain. The workshop was widely advertised across ELIXIR, so the attendees were not only the local researchers based in Finland, but also a few from other Nodes.

The attendees of the hackathon were strongly encouraged to stay for the last two days at the DC workshop as helpers and observers. We did not make it a requirement, since it would make the whole event almost a week long (including the time needed for travel), and may not be possible for some of them to commit to.

***Combined hackathon and workshop in the Netherlands*.** The second event of the Pilot action, hosted by the ELIXIR Netherlands Node, was carried out similarly to the one in Finland. The hackathon and the workshop were co-located and run at the University Medical Centre Utrecht in June 2015. This time 19 participants represented 10 Nodes at the hackathon. To increase the outreach, we encouraged the Nodes to delegate a different representative to the one that was present in Finland - so we had an overlap of only five participants.

The material development took place in three groups:

• Group 1 worked on creating training materials on using ELIXIR Cloud resources;
• Group 2 worked on a decision tree for using cloud computing;
• Group 3 worked on different aspects of understanding how to use one's data for genomics. In particular the group worked on describing the file formats, file manipulation, pipeline integration, post-assembly - *de novo* RNA Transcriptome Analysis, handling blast annotation output and verifying data.

One of the facilitators (and the Pilot proposer) was the same as in Finland. They were joined by another experienced instructor from the US, and a fellow member of the DC Steering Committee. This helped with providing background information to the participants about the workshops and both initiatives. After the hackathon, these instructors taught at the co-located DC workshop that followed and was attended by 30 researchers. Again, the workshop was advertised widely to allow representatives from all ELIXIR Nodes to receive the training. Similarly to the workshop in Finland, the one held at the University Medical Centre Utrecht was also mainly attended by local researchers.

The workshop received a lot of interest, not only from researchers based at universities in the Netherlands and nearby Nodes, but also from industry. One of the companies based in the Netherlands and focusing on bioinformatics contacted the organizers and asked if two of the company researchers could participate in the workshop. As the collaboration with this company is potentially beneficial for both the SWC/DC Foundations and the company, the researchers were welcome to attend. They had a very positive experience and as a result the company requested an internal workshop, which was delivered in December 2015 by two newly trained instructors from the Netherlands eScience Centre.

**Figure 1.**
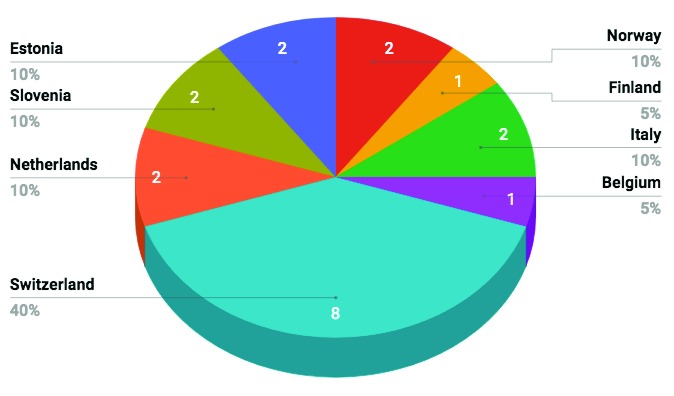
Instructors trained per Node.

***Instructor Training*.** The final event of the Pilot action was the SWC/DC Instructor Training. This covered the process and pedagogy of learning, as well as best practices in teaching, and was not just limited to teaching computational skills. The workshop was delivered over two days and was hosted by the ELIXIR Switzerland Node in Lausanne, Switzerland, in January 2016. Using the communication network we developed during the organisation of the preceding events, we reached out to the ELIXIR Nodes to fill the spaces for the workshop. Eight Nodes were represented at the event and 20 participants were trained as SWC/DC instructors (see Figure 1 below for the spread of participants per Node). As with the previous events, participants had financial support from the Pilot action budget.

This Instructor Training was delivered by two trainers. One of them was the same instructor and facilitator who ran the events in Finland and the Netherlands. This helped with organisation and coordination of the workshop. The second trainer was the Executive Director of the Data Carpentry Foundation, which gave an excellent opportunity for the attendees to discuss various details of planned implementation of DC at their Nodes.

We explicitly advertised the Instructor Training as an event addressed at those representatives of the Nodes who were already interested in training and who aimed to become active Carpentry instructors engaging with the community and running workshops in the future. By the end of 2016, 17 attendees of the Instructor Training have completed the final “Carpentry Instructor Checkout Procedure” and became certified as SWC and/or DC Instructors (out of 20 trained in total - which is a high rate of 85%).

## Main outcomes

Owing to the Pilot action, we trained around 300 researchers and increased the understanding of the SWC/DC training programme, curriculum and model of delivery among the ELIXIR Nodes (see the summary in Table 1). At the beginning of 2015, the Carpentry workshops were known and run primarily in the US, Canada and the UK. By the end of Pilot, the Carpentry programmes are far more known in Europe, and are starting to be endorsed and implemented by an increasing number of Nodes. In total, participants from the following 13 Nodes took part in the Pilot action: Norway, Finland, Italy, Belgium, Switzerland, Netherlands, Slovenia, Estonia, Czech Republic, France, United Kingdom, Israel and Portugal.

**Table 1.** Summary of Pilot action and follow-up activities. Yellow: material creation hackathons, green: workshops, blue: instructor trainings.

Event	Participants, n	ELIXIR Nodes represented, n	Part of the Pilot action?
Material creation hackathon, Finland, March 2015	12	11	Yes
Data Carpentry workshop, Finland, March 2015	29	1	Yes
Material creation hackathon, the Netherlands, June 2015	19	10	Yes
Data Carpentry workshop, the Netherlands, June 2015	30	1	Yes
Instructor Training, Switzerland, January 2016	20	8	Yes
Instructor Training, UCL, UK, October 2016	19	N/A	Pilot follow-up
Instructor Training, Manchester, UK, November 2016	24	N/A	Pilot follow-up
Data Carpentry workshop, Slovenia, July 2016	29	N/A	Pilot follow-up
Software Carpentry workshop, Belgium, November 2016	35	N/A	Pilot follow-up
Software Carpentry workshop, Lausanne, Switzerland, June 2016	30	N/A	Pilot follow-up
Software Carpentry workshop, Basel, Switzerland, June 2016	40	N/A	Pilot follow-up
Data Carpentry workshop, Zurich, Switzerland, July 2016	35	N/A	Pilot follow-up
Data Carpentry workshop, the Netherlands, January 2016	30	N/A	Pilot follow-up
Data Carpentry workshop, Netherlands, April 2016	20	N/A	Pilot follow-up

The training material creation hackathons run during the Pilot allowed the participants to familiarise themselves with the particularities of collaborative material development (one of the main features of SWC/DC). The new materials that were developed (i.e. not contributions to the existing materials) still need to be improved and reviewed. As training develops within ELIXIR, these may become part of the official curriculum, according to the needs of audiences.

The Pilot provided solid foundations for setting up a regular training programme across ELIXIR in computational skills for life sciences. Apart from the growing number of workshops in the UK, Carpentry training events started taking place in other Nodes. Following the Pilot workshops, ELIXIR Slovenia immediately hosted a workshop in July 2015, during which 29 researchers were trained. ELIXIR Belgium organised a workshop in November 2015 and trained 35 researchers. ELIXIR Switzerland organised three workshops: (1) a SWC workshop in Lausanne in June 2016, where 30 researchers were trained; (2) a SWC workshop in Basel in June 2016, where 40 researchers were trained; and (3) a DC workshop in Zurich in July 2016, where 35 researchers were trained. In the Netherlands, 50 researchers were trained in two further workshops, one in January 2016 for Life Scientists and one in April 2016 for the Netherlands Institute for Space Research.

The Pilot action helped in growing the certified SWC/DC instructor pool within the ELIXIR Nodes. It turned out that the demand for Instructor Training from the ELIXIR community was so high that the UK Node, which was the main coordinator of the Pilot, arranged and financed two more Instructor Training events:

• Instructor Training hosted at the University College London in October 2015, during which 19 new instructors were trained;
• Instructor Training hosted at the University of Manchester in November 2015, during which 24 new instructors were trained.

In total, 59 researchers were trained in the Pilot workshops and 221 at follow-up events inspired by the Pilot and funded locally by Nodes. Also, 20 new SWC/DC instructors were trained as part of the Pilot, and 43 at follow-up instructor training events.

## Follow-up

The Pilot action received a lot of interest and positive feedback among the ELIXIR Nodes. We have seen further workshops being hosted within institutions in ELIXIR nodes. The follow-up actions included several wider-scope goals.

ELIXIR and SWC/DC Foundations are finalising (as of summer 2017) the work on a new partnership agreement. SWC/DC run a partnership programme that offers various benefits to organisations choosing different levels of partnering agreements. Due to the size and scope of ELIXIR, the partnership is tailored to the specific needs of the Nodes. The agreement will not only include support for running workshops and instructor trainings, but also assist with developing sustainable training network and possibly incubation of bioinformatics-specific teaching materials.

The ELIXIR Training Coordinators group is looking into integrating Instructor Training into the ELIXIR Train the Trainer programme. Most Nodes already have trainers delivering courses; however, there is still room for growing that pool. SWC/DC Instructor Training may be used as part of professional development - depending on the needs of the specific organisations within ELIXIR.

## Conclusions

The Pilot action was an exercise not only in delivering training, but also in outreaching and disseminating SWC/DC principles in a large-scale international and multi-partner project, such as ELIXIR. The goal was to train researchers in IT skills and introduce SWC/DC workshops across the Nodes. The challenge we faced was to clarify the misconceptions about how the workshops are delivered, their purpose related infrastructure, as well as the application of this training in the life sciences context. We are confident that we have mitigated these risks by bringing in the representatives of the Nodes directly into workshops and hands-on sessions on material development. The participants had a close-up experience of how the workshops operate.

The Pilot helped in forming some suggestions and ideas for possible improvements in developing training programmes.

The engagement of TrCG was essential. In particular, the Coordinators from the Netherlands, Finland and Switzerland (i.e. the Pilot collaborating Nodes) were very helpful. Maintaining this group within ELIXIR is vital for successful growth of its training activities.

However, despite communicating with the TrCG and reaching out to the Nodes through different communication channels during the time period of the Pilot (2015), not all Nodes responded. We have tried to find SWC/DC coordinators in each Node, but possibly it was a bit too early and we will revisit this effort in 2017. Most Nodes did not know enough about the Carpentry trainings and/or do not have a training infrastructure in place (being too small). Furthermore, many Nodes already have their own training programme in place, and it has to be made transparent to them how the SWC/DC training programme can complement that programme.

In 2016, a survey in the ELIXIR nodes was undertaken, and the majority of the nodes did show interest in learning more about SWC/DC and in hosting workshops and hackathons. At this moment, ELIXIR is setting up a collaboration agreement with the SWC/DC Foundations to make it able to roll out workshops over the nodes and, most importantly, to run instructor trainings. In this way, ELIXIR is working towards building a sustainable and self-expanding Carpentry network in Europe.

## Notes

^1^As of November 2015, the ELIXIR Pilot actions have been renamed as Implementation Studies.
