# Supplementary material for: Developing a strategy for computational lab skills training through Software and Data Carpentry: Experiences from the ELIXIR Pilot action
Source: F1000Res. 2017 Jul 3;6:ELIXIR-1040. [Version 1] doi: 10.12688/f1000research.11718.1 (PMC5516217; doi:10.12688/f1000research.11718.1)
Supplement: Peer review history

# Peer review: Referee response for version 1

**DOI:** 10.5256/f1000research.12660.r24016

**Published:** 2017-07-21

**Authors:** Laurent Gatto

**Affiliations:** 1 Computational Proteomics Unit, University of Cambridge, Cambridge, UK

The manuscript describes the efforts within the ELIXIR project to improve teaching of essential data analysis skills that are currently lacking in the research community. To do so, the Pilot action for "Working up and building the foundation for Data Carpentry (DC) and Software Carpentry (SC) within ELIXIR" aims at

- launch DC and SC workshops under the umbrella of ELIXIR
- benefit from the existing qualified instructor pool and support the training of a pool of instructors within the ELIXIR community
- contribute to the DC/SC community in general by growing the number of instructors (point above), and develop or adapt new material dedicated to life sciences.

The manuscript reports on this pilot action. The article also provides a good overview of the interaction between ELIXIR and the Carpentries, and how the latter can contribute to the research community through specific organisations.

 I have two specific suggestions that could possibly further improve the paper.

 Firstly, there are several references to roles without ever specifying who these people were. Some clues are given in the author contributions section, but it would be useful to name those contributors directly. For example in

 "The close collaboration between the UK Node and the SSI substantially facilitated the delivery of the Pilot - one of the key people involved in writing the proposal and delivering the Pilot was the training lead at the SSI at the time, and the deputy head of the UK Node was a co-investigator at the SSI."

 it would be helpful to know who was the person that facilitated the collaboration between the UK Node and SSI.

 Similarly, in

 "We sent a request for volunteers (one per Node) to step in and become a SWC/DC Coordinator for their Nodes."

 It would be useful the name those volunteers.

 Getting credit for such efforts is important to support the kind of activities that are promoted by the pilot action, as it relies, as are Carpentries workshops themselves, on volunteering. In addition, I feel that being explicit as to whom did what would help consolidating a wider community around the effort.

 My second point relates to the assessment of success, both in terms of new instructors and participants. Do the authors have data on whether the newly trained instructors have they been helpers, (lead) instructors or organised workshops themselves, and have they been able to follow up with workshop participants to assess to what extend they apply what they have learnt.

 I spotted one typo in the introduction: "It coordinates and sustains bioinformatics resources across its member states and help[s] researchers ...".

I have read this submission. I believe that I have an appropriate level of expertise to confirm that it is of an acceptable scientific standard.

# Peer review: Referee response for version 1

**DOI:** 10.5256/f1000research.12660.r24013

**Published:** 2017-07-17

**Authors:** Anelda P. van der Walt, Juan Steyn

**Affiliations:** 1 Talarify (PTY) LTD, Potchefstroom, South Africa; 2 North-West University, Potchefstroom, South Africa

The article provides a good overview of a pilot project that was run through ELIXIR to develop a training program to teach foundation computing and data skills to researchers.

 The authors give context of ELIXIR's mission and aims and how the pilot was put together and rolled out. It also provides information about the pilot's impact, specifically mentioning subsequent activities that took place because of the project.

 Given that this is an opinion article, it would be interesting to learn more about what ELIXIR did before employing the Carpentries model in terms of computational and data training. What other training platforms or methodologies have they tried and how do the Carpentries compare to other models they've employed. It would also be very useful if the authors could explain exactly what aspects of the Carpentries appealed to ELIXIR specifically and caused them to adopt it during the pilot. They did describe both Software and Data Carpentry, but it would be useful to explicitly describe why the Carpentries had such an appealing fit for ELIXIR’s need. There are many organisations these days that resemble the ELIXIR structure of hub and nodes, not only in the life sciences but also in nanoscience, mathematics and statistics, and other areas. This kind of information may help those who are not familiar with the Carpentries to evaluate whether a similar approach for developing computational capacity in their organisations may be relevant to their specific contexts.

 In some cases the authors use words such as "community of experts" and "experienced instructor", "perfected". Two of the core philosophies of the Carpentries are openness and collaboration. Although the Carpentries include a large number of experts and experienced instructors, the community embraces anyone who can contribute in any way and is by no means an elitist club of experts. In many cases lesson contributions are made by learners or newly qualified instructors. The Carpentries is specifically open and welcoming to anyone, not only the experts. The quality of the lesson material could equally be attributed to the collaborative development process, peer review, and repetitive use in workshop settings, and to leadership in its development by experts who often also play the role of lesson maintainers. The Carpentries are ever-evolving organisations that constantly learn from their community of instructors and learners, as well as research about learning and teaching in order to offer more relevant training, to improve the lesson material, the format of the workshops, and the teaching methodologies. Instead of describing workshops as having been "perfected" it could potentially rather be described as "constantly evolving to incorporate the latest research on teaching and learning" or something to this effect.

 The authors could reference the original Data Carpentry publication where it indicates that Data Carpentry started running workshops in 2014 (http://ijdc.net/index.php/ijdc/article/view/10.1.135/386).

 The authors should note that SQL was removed as a core topic from the Software Carpentry curriculum in May 2015 (https://software-carpentry.org/blog/2015/03/and-now-we-are-three.html).

 Another interesting datapoint might be to show how many of the newly trained instructors delivered the training that followed after completion of the pilot action. Were ELIXIR members teaching ELIXIR workshops? Were they teaching at non-ELIXIR workshops as one of the outcomes of the pilot was to help build instructors that could serve the broader community? The authors specifically stated that this was one of the intentions of the pilot as well.

 In the section under “Outreach”, the authors mention that the Pilot commenced in late 2014 but under the section “Pilot action overview and goals” it is stated that the pilot ran from March 2015. Could the authors please clarify?

 In a study done by Beth Duckles in 2015 Carpentry instructors were asked what personal and professional benefits were associated with being involved in the Carpentries. This report is a valuable reference to provide more clarity on the reason for instructors volunteering to teach. A number of reasons for teaching were listed including but not limited to (1) having the opportunity to travel, (2) building their resumé (teaching experience), (3) enjoying being part of a community, (4) improving their teaching skills and learning from other instructors, (5) learning to communicate and present more efficiently, and more. The authors may want to provide more concrete reasons for why instructors teach and reference the report available at https://software-carpentry.org/files/bib/duckles-instructor-engagement-2016.pdf

 Moving the section on Software and Data Carpentry before the section about ELIXIR and the Carpentries may make more sense logically.

We have read this submission. We believe that we have an appropriate level of expertise to confirm that it is of an acceptable scientific standard.

## References

[1] TealTCranstonKLappHWhiteEWilsonGRamKPawlikA: Data Carpentry: Workshops to Increase Data Literacy for Researchers. *International Journal of Digital Curation*.2015;10(1) : 10.2218/ijdc.v10i1.351 10.2218/ijdc.v10i1.351

# Peer review: Referee response for version 1

**DOI:** 10.5256/f1000research.12660.r24015

**Published:** 2017-07-17

**Authors:** Erin A. Becker

**Affiliations:** 1 Data Carpentry, San Francisco Bay, CA, USA

This is an interesting article presenting a process for implementing the SWC/DC model of instructor training to a large, pan-European research community. 

 One statement should be corrected. The description of DC says that "DC designs the workshops to fit into needs of particular domains (e.g. life sciences, social sciences, digital humanities and library carpentries, etc.)." This is inaccurate as DC materials do not currently exist for digital humanities or social sciences (although social sciences materials are in progress and plans for digital humanities curricula are in discussions). Additionally, library materials are managed under Library Carpentry as an independent organization.

I have read this submission. I believe that I have an appropriate level of expertise to confirm that it is of an acceptable scientific standard.
